# IMM2510, a novel anti-PD-L1/VEGF bispecific antibody for cancer immunotherapy

**DOI:** 10.1093/abt/tbag002

**Published:** 2026-01-15

**Authors:** Dianze Chen, Zhuli Wu, Xiwen Zhao, Rupal S Bhatt, Yanan Yang, Wenqi Zhu, Huang Tang, Kaili Wang, Chunli Guo, Dandan Liu, Chunmei Yang, Huiqin Guo, Xing Bai, Ruliang Zhang, Song Li, Wenzhi Tian

**Affiliations:** ImmuneOnco Biopharmaceuticals (Shanghai) Inc., Shanghai 201203, China; ImmuneOnco Biopharmaceuticals (Shanghai) Inc., Shanghai 201203, China; ImmuneOnco Biopharmaceuticals (Shanghai) Inc., Shanghai 201203, China; Instil Bio Inc., 3963 Maple Ave Ste 350, Dallas, TX 75219, United States; ImmuneOnco Biopharmaceuticals (Shanghai) Inc., Shanghai 201203, China; ImmuneOnco Biopharmaceuticals (Shanghai) Inc., Shanghai 201203, China; ImmuneOnco Biopharmaceuticals (Shanghai) Inc., Shanghai 201203, China; ImmuneOnco Biopharmaceuticals (Shanghai) Inc., Shanghai 201203, China; ImmuneOnco Biopharmaceuticals (Shanghai) Inc., Shanghai 201203, China; ImmuneOnco Biopharmaceuticals (Shanghai) Inc., Shanghai 201203, China; ImmuneOnco Biopharmaceuticals (Shanghai) Inc., Shanghai 201203, China; ImmuneOnco Biopharmaceuticals (Shanghai) Inc., Shanghai 201203, China; ImmuneOnco Biopharmaceuticals (Shanghai) Inc., Shanghai 201203, China; ImmuneOnco Biopharmaceuticals (Shanghai) Inc., Shanghai 201203, China; ImmuneOnco Biopharmaceuticals (Shanghai) Inc., Shanghai 201203, China; ImmuneOnco Biopharmaceuticals (Shanghai) Inc., Shanghai 201203, China

**Keywords:** bispecific antibody, VEGF, PD-L1, ADCC, ADCP, immunotherapy

## Abstract

**Background:**

Dual inhibition of PD-1/PD-L1 and VEGF/VEGFR pathways is a promising strategy to overcome tumor immune evasion and inhibit angiogenesis. IMM2510 is a novel PD-L1 × VEGF bispecific antibody, constructed by fusing VEGFR1 domain 2 (VEGFR1D2) to each anti-PD-L1 heavy chain. In addition, IMM2510 incorporates an Fc region engineered for enhanced antibody-dependent cellular cytotoxicity (ADCC), enabling elimination of PD-L1-expressing tumor and stromal cells.

**Methods:**

Binding and blocking activities were assessed using enzyme-linked immunosorbent assay, surface plasmon resonance, and flow cytometry. Functional assays included Jurkat-PD-1 and VEGFR2 reporter systems, HUVEC proliferation, mixed lymphocyte reaction, and NK cell-mediated cytotoxicity. Cooperative binding with VEGF165 was evaluated biochemically and in reporter assays. Antitumor efficacy was tested in MC38-hPD-L1 syngeneic tumors, HCC827 non-small cell lung cancer (NSCLC) xenografts, and MDA-MB-231 triple-negative breast cancer (TNBC) xenografts.

**Results:**

IMM2510 bound PD-L1, VEGF-A, VEGF-B, and PlGF with high affinity, and blocked both PD-1/PD-L1 and VEGF/VEGFR interactions. It reversed PD-1-mediated T-cell inhibition, inhibited VEGF-driven endothelial proliferation, and induced potent ADCC and ADCP in killing PD-L1^+^ tumor cells. Preincubation with VEGF165 enhanced PD-L1 binding and checkpoint blockade activity, indicating cooperative binding. *In vivo*, IMM2510 induced dose-dependent tumor growth inhibition, achieving superior efficacy to parental monotherapies and their combination. Consistent efficacy was observed across multiple tumor types, including NSCLC and TNBC.

**Conclusions:**

IMM2510 combines checkpoint blockade, anti-angiogenesis, Fc-mediated effector function, and cooperative binding, resulting in superior preclinical antitumor activity across diverse tumor settings. These findings position IMM2510 as a differentiated next-generation therapeutic candidate for clinical development.

## Teaser

**Statement of Significance** MM2510 is an Fc-engineered PD-L1 × VEGF bispecific antibody in which each anti-PD-L1 heavy chain is fused to VEGFR1 domain 2. Preclinical studies have characterized the binding, functional activity, and antitumor efficacy, thus supporting it as a promising next-generation therapeutic candidate with broad potential in solid tumors.

## Background

Immune checkpoint inhibitors (ICIs) targeting the PD-1/PD-L1 axis have transformed the treatment landscape for multiple cancers, producing durable responses in a subset of patients [1–3]. However, the majority of patients either fail to respond or eventually develop resistance [4]. One contributing factor is the immunosuppressive tumor microenvironment (TME), in which vascular endothelial growth factor (VEGF) plays a pivotal role [5–7]. Beyond its well-known pro-angiogenic function, VEGF can inhibit dendritic cell maturation, promote regulatory T-cell (Treg) expansion, and impair T-cell trafficking into tumors [8–10].

Therapeutic blockade of VEGF/VEGFR signaling has demonstrated antitumor activity and, in combination with ICIs, has yielded improved survival outcomes in several malignancies, including non-small cell lung cancer (NSCLC), hepatocellular carcinoma (HCC), and renal cell carcinoma [11–14]. The clinical success of combinations such as atezolizumab plus bevacizumab underscores the complementary nature of VEGF inhibition and PD-L1 blockade [12, 13]. Nevertheless, co-administration of two monoclonal antibodies increases treatment complexity, cost, and the risk of additive toxicities [15].

Bispecific antibodies (bsAbs) represent an innovative therapeutic format capable of targeting multiple pathways simultaneously within a single molecule [16, 17]. This approach offers several potential advantages over combination therapy with two separate antibodies: simplified dosing, improved pharmacokinetics, optimized target engagement, and the opportunity to engineer unique functional properties, such as cooperative binding or enhanced Fc-mediated effector functions [16, 18].

PD-(L)1 × VEGF bsAbs are an emerging subclass within this category, with agents such as ivonescimab [19–21] and BNT327/PM8002 [22–24] already in clinical development. Early-phase studies of these molecules have shown encouraging antitumor activity and tolerable safety profiles across multiple tumor types, reinforcing the therapeutic potential of this dual-targeting strategy.

IMM2510 is a novel PD-L1 × VEGF bispecific IgG1 antibody in which each anti-PD-L1 heavy chain is N-terminally fused to a VEGFR1 domain 2 (VEGFR1D2), enabling simultaneous blockade of PD-1/PD-L1 and VEGF/VEGFR signaling. Importantly, IMM2510 is, to our knowledge, the only molecule in this class incorporating an Fc domain engineered for enhanced antibody-dependent cellular cytotoxicity (ADCC). This design feature provides the ability to deplete PD-L1-expressing tumor and stromal cells in addition to checkpoint blockade and anti-angiogenesis. Given the role of PD-L1-expressing cells in maintaining an immunosuppressive TME, we hypothesize that Fc-enhanced ADCC may be a critical contributor to efficacy, offering a differentiated therapeutic profile compared with existing PD-L1 × VEGF bsAbs.

Preclinical studies were undertaken to characterize the binding, functional activity, and antitumor efficacy of IMM2510, and to explore unique mechanistic features such as VEGF-enhanced cooperative binding to PD-L1, a property that not only underscores the structural synergy of this bispecific design but also suggests a potential contribution to its heightened biological potency and antitumor efficacy.

## Materials and methods

### Cell culture

The Jurkat, RKO, Raji, HCC827, ES-2, HT-1080, 5637 and SK-N-SH cell lines were purchased from the Cell Bank of the Chinese Academy of Sciences. FcγRIIIA (158 V)-NK92MI (FcR-TANK), Jurkat-NFAT-eGFP-PD1, Raji-PDL1 and 293 T-NFAT-eGFP-VEGFR2 were generated in-house. Jurkat, Raji, HCC827 and 5637 cells were incubated at 37 °C in 5% CO_2_ and cultured in RPMI 1640 medium (Gibco, Cat# 11875093) supplemented with 1% penicillin–streptomycin (PS) (Gibco, Cat# 15140122) and 10% fetal bovine serum (FBS) (Gibco, Cat#10091148). RKO, HT-1080, SK-N-SH and 293 T-NFAT-eGFP-VEGFR2 were cultured in DMEM medium (Gibco, Cat# 11965092) supplemented with 1% PS and 10% FBS. ES-2 was cultured in McCoy’s 5A medium (Gibco, Cat# 16600082) supplemented with 1% PS and 10% FBS. FcR-TANK was cultured in serum-free TANK medium (ImmuneOnco, Cat#CT001–1).

### Recombinant proteins and antibodies

The recombinant proteins used included: Human VEGF-A (VEGF165) (Sino Biological, Cat#11066-HNAH), Human VEGF-B (Acro Biosystems, Cat#VE6-H5225), Human VEGF-C (Sino Biological, Cat# 10542-H08H), Human VEGF-D (Sino Biological, Cat# 10557-H08H), Human PIGF (Sino Biological, Cat# 10274-HNAE1), Mouse VEGFA (Sino Biological, Cat#50159-HNAB), Rat VEGFA (Sino Biological, Cat#80006-RNAB), Human PD-L1 His (Sino Biological, Cat#10084-H08H), Human PD-L2 His (Sino Biological, Cat#10292-H08H), Cynomolgus PD-L1 His (Sino Biological, Cat#90251-C08H), Mouse PD-L1 His (Sino Biological, Cat#50010-M08H), Rat PD-L1 His (Sino Biological, Cat#80450-R08H).

IMM2510 is a bispecific antibody targeting both VEGF and PD-L1. Briefly, the extracellular 2nd domain of human VEGFR1 (VEGFR1D2) was fused to the N-terminus of both heavy chains of the anti-PD-L1 antibody. The antibodies, including IMM2510, IMM25 and IMM25-N297A (anti-PD-L1 antibody), VEGFR1-Fc, and hIgG1-Fc (isotype control) were produced in-house. Atezolizumab was purchased from Genentech, ivonescimab from Akesobio and bevacizumab from Roche.

### Binding kinetic analysis

The binding affinity of IMM2510 to human PD-1 and VEGF were determined by surface plasmon resonance, SPR device (Biacore T200, GE Healthcare). 20 μg/ml of anti-human IgG (Fc) antibody was immobilized to CM5 chip, and then 5 μg/ml of IMM2510 was captured. The serial dilution of human PD-L1 (Sino Biological, Cat#10084-H08H-100) or VEGF165 (Sino Biological, Cat#11066-HNAH) were injected over the sensor chip at 30 μl/min. The data were analyzed and the binding affinity (KD value) was calculated using the Biacore T200 Evaluation Software v.3.1.

The Gator™ Label-Free Bioanalysis instrument Gator Bio was used to detect the simultaneous binding kinetics of IMM2510 to VEGF and PD-L1. The anti-human IgG probes (Gator Bio, Cat#20–5036) were pre-incubated for 120 s in Q buffer (10 mM PBS, 0.02% tween, 0.2% BSA; pH 7.4). 10 μg/ml IMM2510 was captured by the anti-human IgG Fc probes and the probes were incubated with VEGF165, then with PD-L1-His or in the reverse order, blank buffer was used as negative control. The data were analyzed using the Gator software.

### Enzyme-linked immunosorbent assay

For binding enzyme-linked immunosorbent assays (ELISAs), 96-well plates were coated with 100 ng/well of human PD-L1 or VEGF overnight at 4 °C. The coated plates were blocked with 3% skim milk in PBST and then serial dilutions of IMM2510 or a hIgG1 isotype control were added and incubated for 1 h. After washing with PBST five times, peroxidase-conjugated anti-human IgG secondary antibody (Jackson Immuno, Cat#109–006-008) was added and incubated for 1 h. After incubation, the plates were washed again and The substrate solution TMB (KPL, Cat#51200050) was added for the color reaction. The reaction was terminated with 2 M sulfuric acid and absorbance was measured at 450 nm using the microplate reader (BioRad, iMARK).

### Simultaneous binding by ELISA

To evaluate simultaneous binding, plates were coated with 100 ng/well human PD-L1-hFc and then serial dilutions of IMM2510 were added. Following incubation and washing, biotinylated human VEGF-hFc was added. HRP-conjugated streptavidin (Cell Signaling, Cat#3999S) was added and incubated. Detection was performed as described in section 2.4.

### Blocking assay by ELISA

To assess blockade of VEGF/VEGFR1 interaction, plates were coated with human VEGF165. After blocking, serial dilutions of IMM2510 were added. Subsequently, biotinylated human VEGFR1-hFc was added, followed by HRP-conjugated streptavidin. Detection was performed as described above.

### Binding assays by flow cytometry

Cell-based binding was evaluated on PD-L1^+^ tumor cells. Serial dilutions of IMM2510 were incubated with tumor cells (5 × 10^5^ cells/ml) at 4 °C for 45 min. After washing with PBS containing 1%BSA, cell-bound antibodies were detected using a FITC-conjugated anti-human IgG Fc (Sigma Aldrich, Cat# F9512). Fluorescence was measured using flow cytometry (Luminex, Guava® easyCyte™ 8HT System) and mean fluorescence intensity was analyzed using GuavaSoft software v3.0.

### Blocking assay by flow cytometry

To assess blockade of PD1/PD-L1 interaction, serial dilutions of IMM2510 were incubated with PDL1-mFc (3 μg/ml) at 4 °C for 45 min and then CHO-PD1 cells (5 × 10^5^ cells/ml) were added. After incubation, cell-bound PDL1-mFc was detected using a PE-conjugated minimal x-reactivity anti-mouse IgG (Biolegend, Cat#405307). Fluorescence was measured and analyzed as in section 2.7.

### PD-1/PD-L1 blockade reporter gene assay

The Jurkat-NFAT-eGFP-PD1 Reporter Cell line was engineered to stably express human PD-1 and the NFAT response element driving eGFP expressing systems. When co-cultured with Raji-PDL1, the PD-1/PDL1 interaction inhibits TCR signaling and NFAT-mediated eGFP fluorescence. IMM2510 blocks this interaction, thereby restoring NFAT activation and eGFP reporter expression. Briefly, Jurkat-NFAT-eGFP-PD1 cells (3 × 10^5^ cells/ml) and Raji-PDL1 cells (6 × 10^5^ cells/ml) were harvested and seeded into 96-well plates. Serially diluted antibodies were added, and the plates were incubated overnight at 37 °C in 5% CO_2_. The eGFP fluorescence signals in Jurkat cells were assessed using flow cytometry (Luminex, Guava® easyCyte™ 8HT System) and the mean fluorescence intensity was analyzed using GuavaSoft software v3.0.

### VEGF reporter gene assay

The 293 T-NFAT-eGFP-VEGFR2 reporter cell line was engineered to stably express human VEGFR2 and the NFAT response element driving eGFP expression. VEGF165 binding to VEGFR2 activates the VEGFR2 signaling pathway, leading to NFAT activation and eGFP expression. Briefly, 50 ng/ml VEGF165 was pre-incubated with serially diluted antibodies for 45 min and then was added to a 96-well plate. 293 T-NFAT-eGFP-VEGFR2 cells (3 × 10^5^ cells/ml) were seeded and incubated overnight at 37 °C in 5% CO_2_. eGFP fluorescence was assessed by flow cytometry, and the mean fluorescence intensity was analyzed.

### HUVEC proliferation assay

Human umbilical vein endothelial cells, HUVEC (Allcells, Cat#HUVEC-001F), were cultured at 37 °C in 5% CO_2_ in a complete HUVEC medium (Allcells, Cat# H-004). For the proliferation assay, HUVEC were seeded into 96-well plates with 3000 cells/well and incubated for 24 h at 37 °C in 5% CO_2_. The culture medium was replaced with RPMI 1640 (Gibco, Cat#11875093) supplemented with 2% heat-inactivated FBS. 100 ng/ml VEGF165 preincubated with serially diluted antibodies for 45 min, was added. Following a 3-day incubation, 20 μl Cell Counting Kit-8 (Beyotime, Cat# C0040) was added and incubated for 2 h, the absorbance was measured at 450 nm using the microplate reader (BioRad, iMARK).

### Mixed lymphocyte reaction assay

CD4^+^ T cells were isolated from human PBMCs using the EasySep™ Human CD4^+^ T Cell Isolation Kit (Stemcell, Cat# 17952). Mature dendritic cells (mDCs) were isolated and induced from human PBMCs using the EasySep™ Human Monocyte Isolation Kit (Stemcell, Cat# 19359). CD4^+^ T cells (2 × 10^5^ cells/ml) and mDCs (2 × 10^4^ cells/ml) were co-cultured in 96-well plates. Serially diluted antibodies were added and incubated overnight at 37 °C in 5% CO_2_ for 4 days. Supernatants were collected, and IL-2 and IFN-γ were quantified using the CBA Human IL-2 (BD, Cat#558270) and IFN-γ (BD, Cat#558269) kits.

### ADCC and ADCP assay

**ADCC assay:** the target cells labeled with CSFE (Sigma, Cat#21888) and FcγRIIIA (158 V)-NK92MI (FcR-TANK™) cells were seeded into 96-well plates. Serially diluted antibodies were added, and incubated at 37 °C in 5% CO2 for 4 h. Target cell killing was assessed by flow cytometry after labeling with propidium iodide (Sigma, Cat#P4170), or by measuring absorbance at 450 nm using the Cell Counting Kit-8.

**ADCP assay:** THP-1 cells (2 × 10^5^ cells/ml) harvested and seeded into 96-well plates, then incubated for 20 h with 200 ng/ml PMA (Sigma, Cat#79346) and 60 ng/ml IFN-γ (Sino Biological, Cat#11725-HNAS). Target cells (5 × 10^5^ cells/ml) labeled with CSFE and serially diluted antibodies were added and incubated at 37 °C in 5% CO_2_ for 2 h. After washing to remove non-phagocytosed targets, the CFSE fluorescence in THP-1 cells was measured by flow cytometry, and the mean fluorescence intensity was analyzed.

For the ADCP reporter gene assay, Jurkat-NFAT-Luc-CD64 reporter cells were engineered to stably express human CD64 and the NFAT response element driving luciferase expressing systems. Briefly, Jurkat-NFAT-Luc-CD64 (4 × 10^5^ cells/ml) and Raji-PDL1 cells (2 × 10^5^ cells/ml) were seeded into 96-well plates. Serially diluted antibodies were added into the plates, and incubated overnight at 37 °C in 5% CO_2_. Steady-Lumi™ II Firefly Luciferase assay reagent (Beyotime, Cat#RG059M) was added, and luminescence was quantified by SpectraMax M3 (Molecular Devices).

### Sec-HPLC

Human VEGF165 and the complexes of antibody (1000 nM) and VEGF165 (1000 nM) at 1:1 molar ratio were analyzed for SEC-HPLC using Waters ACQUITY Arc. 100 μl mixed samples were injected separately into a pre-equilibrated XBridge BEH200A SEC (Waters) 3.5 μm size exclusion column. The mobile phase was 10 mM phosphate, 300 mM NaCl, pH 7.2 with a flow rate of 0.5 ml/ min.

### *In vivo* anti-tumor activity studies

**Raji-PDL1 xenograft tumor model in CB17-SCID mice:** Raji-PDL1 cells were resuspended in PBS and implanted subcutaneously at 5 × 10^6^ cells/mouse in the right flank of CB17-SCID mice. The mice were randomly divided into three groups (n = 6 per group): PBS, IMM25-N297A (5 mg/kg), and IMM25 (5 mg/kg). The antibodies were injected intraperitoneally twice weekly. The tumor volume and animal body weight were measured three times weekly using a digital caliper, and the volume was calculated using the formula: TV = 0.5 × a × b^2^, where a and b are the longest and shortest tumor diameters, respectively.

**MC38-hPD-L1 syngeneic tumor model in hPD-1-Tg C57BL/6 mice:** Mouse colon cancer MC38-hPD-L1 cells were resuspended in PBS and implanted subcutaneously at 3 × 10^5^ cells/mouse in the right flank of hPD-1-Tg C57BL/6 mice. When the mean tumor volume reached ~73 mm^3^, mice were randomly divided into seven groups (n = 10 per group): PBS, atezolizumab (5 mg/kg), VEGFR1-Fc (1 mg/kg), IMM2510 (2 mg/kg), IMM2510 (6 mg/kg), IMM2510 (12 mg/kg), VEGFR1-Fc + atezolizumab (1 + 5 mg/kg). Then the antibodies were injected intraperitoneally twice weekly. The tumor volume and animal body weight were measured three times weekly. Tumor growth inhibition rate (TGI) was calculated using the formula: TGI = (1-T/C) × 100%, where T is the average relative tumor volume (RTV) of the treated group, and C is the vehicle control group. RTV is the post-treatment tumor volume relative to pre-dose.

**HCC827 xenograft tumor model in Balb/c nude mice:** HCC827 cells were resuspended in PBS, mixed with Matrigel (v/v = 1:1) and implanted subcutaneously at 1 × 10^7^ cells/mouse in the right flank of Balb/c nude mice. When the mean tumor volume reached ~139 mm^3^, mice were randomly divided into six groups (n = 6 per group): PBS, VEGFR1-Fc (2 mg/kg), atezolizumab (6 mg/kg), IMM25 (6 mg/kg), IMM2510 (8 mg/kg), VEGFR1-Fc + IMM25 (2 + 6 mg/kg). Then the antibodies were injected intraperitoneally twice weekly. The tumor volume and animal body weight were measured twice weekly.

**MDA-MB-231 xenograft tumor model in Balb/c nude mice:** MDA-MB-231 cells were resuspended in PBS and mixed with Matrigel (v/v = 1:1) and implanted subcutaneously at 1 × 10^7^ cells/mouse in the right flank of Balb/c nude mice. When the mean tumor volume reached ~108 mm^3^, mice were randomly divided into six groups (n = 6 per group): PBS, VEGFR1-Fc (2 mg/kg), Atezolizumab (6 mg/kg), IMM25 (6 mg/kg), IMM2510 (8 mg/kg), VEGFR1-Fc + IMM25 (2 + 6 mg/kg). Then the antibodies were injected intraperitoneally twice weekly. The tumor volume and animal body weight were measured twice weekly.

### Statistical analysis

All statistical analyses were conducted using GraphPad Prism 10.6 (GraphPad Software, Inc.). Differences among three or more groups were assessed by one-way ANOVA, while intergroup differences were evaluated by Student’s T-tests. Significance levels were defined as follows: ns, not significant (*P* > .05); ^*^P ≤ .05; ^**^P ≤ .01; ^***^P ≤ .001; ^****^P ≤ .0001.

## Results

### Structure and binding activity of IMM2510

**Figure 1:**
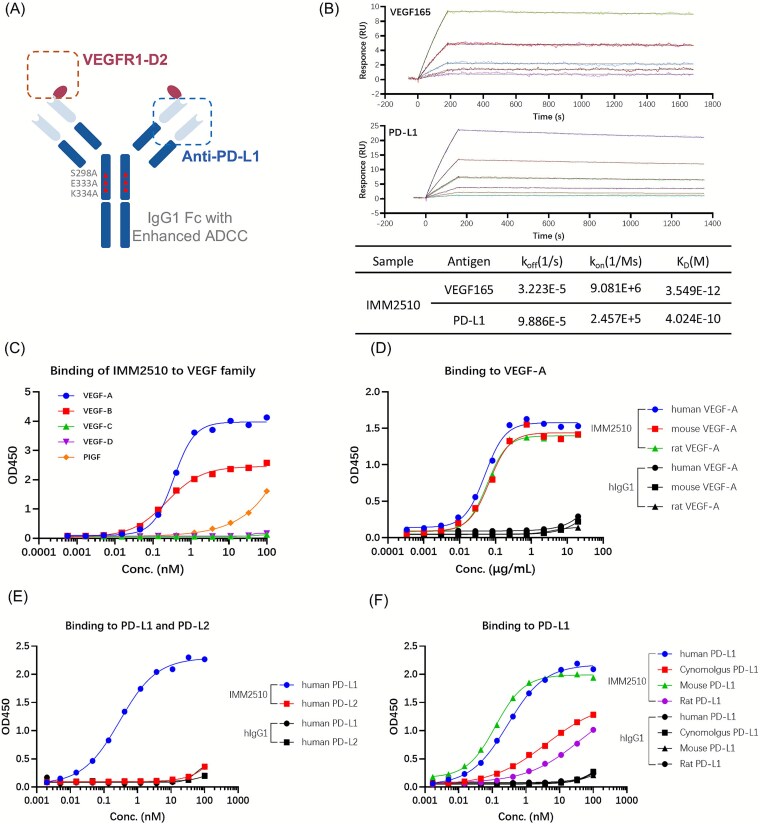
Structure and binding activity of IMM2510. (a) Structure diagram of IMM2510; (b) binding affinity of IMM2510 to human VEGF165 and PD-L1 measured by SPR; (c) binding of IMM2510 to VEGF family ligands; (d) cross-species binding of IMM2510 to VEGF; (e) specificity of IMM2510 binding to human PD-L1 and PD-L2; (f) cross-species binding of IMM2510 to PD-L1;

IMM2510 is a fully humanized bispecific IgG1 antibody in which each anti-PD-L1 heavy chain is N-terminally fused to VEGFR1 domain 2 (VEGFR1D2). Mutations of S298A/E333A/K334A in Fc region were introduced to enhance Fc-mediated ADCC (Fig. 1a). Notably, the triple alanine mutant S298A/E333A/K334A is known to improve IgG1 affinity for FcγRIIIA [25]. This structure exhibits concurrent engagement of PD-L1 and VEGF family ligands, including VEGF-A, VEGF-B, and PlGF (Fig. 1c) and has cross-reactivity to rat and mouse VEGF-A (Fig. 1d). Surface plasmon resonance (SPR) analysis demonstrated that IMM2510 binds to recombinant human PD-L1 (3.549 × 10^−12^ M) and VEGF165 (4.024 × 10^−10^ M) with dissociation constants in the sub-nanomolar range (Fig. 1b). Furthermore, IMM2510 bound specifically to human PD-L1 but not to human PD-L2 (Fig. 1e) and showed cross-reactivity to cynomolgus, rat and mouse PD-L1 (Fig. 1f). In cell-based binding assay, IMM2510 bound to PD-L1+ tumor cells (Supplementary Fig. S1). These data demonstrate that the fusion of VEGFR1D2 does not impair PD-L1 binding, and Fc engineering does not alter ligand recognition.

### IMM2510 blocks both VEGF/VEGFR and PD-1/PD-L1 interactions

**Figure 2:**
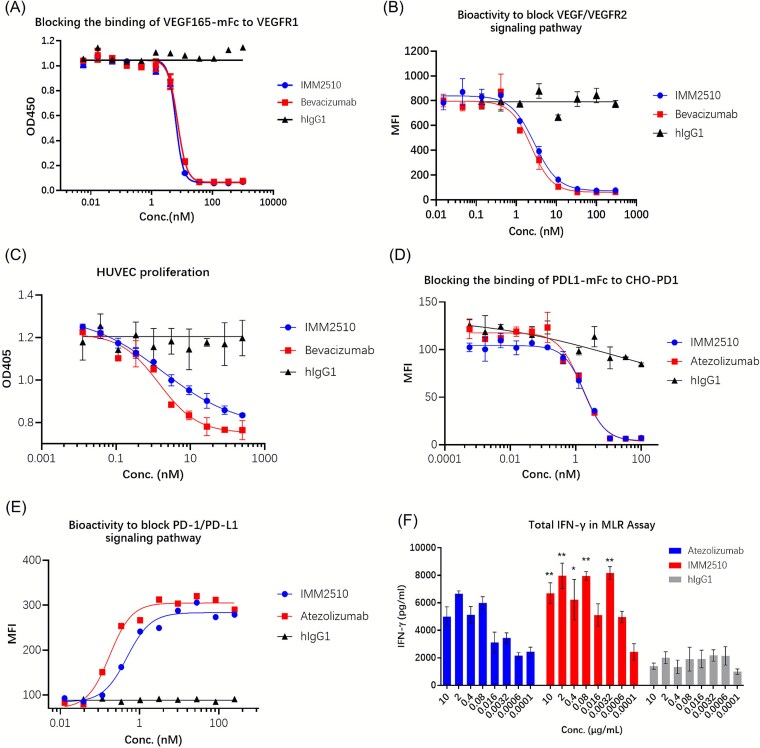
IMM2510 blocks both VEGF/VEGFR and PD-1/PD-L1 interactions. (a) Blockade of VEGF165-mFc binding to VEGFR1 by ELISA; (b) inhibition of VEGF/VEGFR2 signaling in 293 T-NFAT-eGFP-VEGFR2 reporter assay; (c) inhibition of VEGF-induced HUVECs proliferation; (d) blockade of PDL1-mFc binding to CHO-PD1 cells by flow cytometry; (e) inhibition of PD1/PD-L1 signaling in Jurkat-NFAT-eGFP-PD1 reporter assay; (f) dose-dependent increase in IFN-γ release of in MLR assay, ^*^P < .05, ^**^P < .01, ^***^P < .001 vs. hIgG1 isotype control.

The ability of IMM2510 to inhibit both target pathways was next assessed. In VEGF165/VEGFR1 blocking and VEGF165/VEGFR2 reporter assay, IMM2510 prevented ligand-receptor binding with potency comparable to bevacizumab (Fig. 2a and b). Furthermore, IMM2510 significantly inhibited HUVEC proliferation (Fig. 2c). Similarly, in the PD-1/PD-L1 blocking assay by flow cytometry, IMM2510 effectively blocked PD-L1-mFc binding to PD-1-expressing cells in a dose-dependent manner, achieving near-complete inhibition comparable to atezolizumab (Fig. 2d). Functional blockade was confirmed in a PD-1 reporter assay, where IMM2510 disrupted the PD-1/PD-L1-mediated suppression of TCR signaling in Jurkat-NFAT-eGFP-PD-1/Raji-PD-L1 co-cultures, resulting in a dose-dependent increase in NFAT-driven eGFP fluorescence (Fig. 2e). Moreover, in mixed lymphocyte reaction (MLR) assay IMM2510 significantly increased IFN-γ release in a dose-dependent manner (Fig. 2f), indicating the reversal of PD-1-mediated T-cell inhibition. These findings demonstrate that IMM2510 effectively preserves the functional activity of each parental arm, thus demonstrating potent blockade of both immune checkpoint signaling and VEGF/VEGFR1-mediated angiogenesis.

### IMM2510 induces tumor cell lysis via ADCC and ADCP

**Figure 3:**
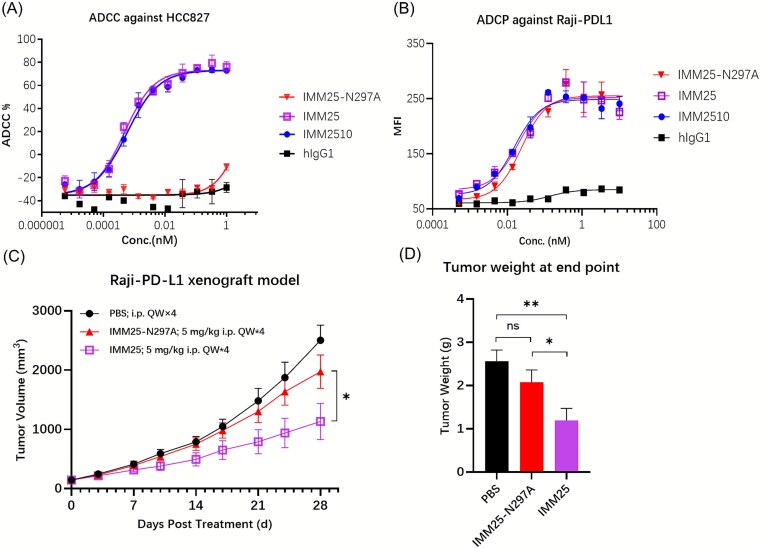
IMM2510 induces tumor cell lysis via fc-mediated ADCC and ADCP. (a) ADCC activity against HCC827 cells co-cultured with FcγRIIIA (158 V)-NK92MI effector cells for 24 h, measured by cell counting Kit-8; (b) ADCP activity against CFSE-labeled Raji-PD-L1 cells co-cultured with PMA-differentiated THP-1 macrophages for 2 h, measured by flow cytometry; (c) tumor growth curves in CB17-SCID mice bearing Raji-PDL1 xenograft; (d) terminal tumor weights. ^*^P < .05, ^**^P < .01, ^***^P < .001;

To assess the biologic effects of the Fc region engineering for enhanced FcγRIIIa binding, we evaluated and compared the capacity of IMM2510 to mediate antibody-dependent cellular cytotoxicity (ADCC) with that of IMM25-N297A, an in-house generated anti-PDL1 antibody with the N297A mutation in the Fc region. As shown in Fig. 3a and Supplementary Fig. S2a, in FcγRIIIA (158 V)-NK92MI cells-mediated cytotoxicity assays, IMM25 (a PD-L1 antibody sharing the same mutations of S298A/E333A/K334A as IMM2510 but lacking VEGFR1D2) induced lysis of PD-L1-expressing HCC827 target cells, whereas IMM25-N297A did not. IMM2510 displayed ADCC activity comparable to IMM25, confirming that VEGFR1D2 fusion does not impair Fc effector function. Additionally, IMM2510 and IMM25 demonstrated antibody-dependent cellular phagocytosis (ADCP) activity similar to IMM25-N297A (Fig. 3b and Supplementary Fig. S2b).

The contribution of Fc modifications to *in vivo* efficacy was evaluated in CB17-SCID mice bearing Raji-PD-L1 xenografts, IMM25 exhibited significantly greater anti-tumor activity than IMM25-N297A (Fig. 3c and d), indicating that the ADCC-enhanced Fc modification contributes substantially to the *in vivo* antitumor efficacy, highlighting ADCC as a critical mechanism of action for anti-PD-L1 mAbs. These results establish the unique capacity of IMM2510 to deplete PD-L1-expressing tumor and stromal cells via ADCC, in addition to its checkpoint blockade function.

### VEGF165 enhances the binding and blocking activity of IMM2510 toward PD-L1

**Figure 4:**
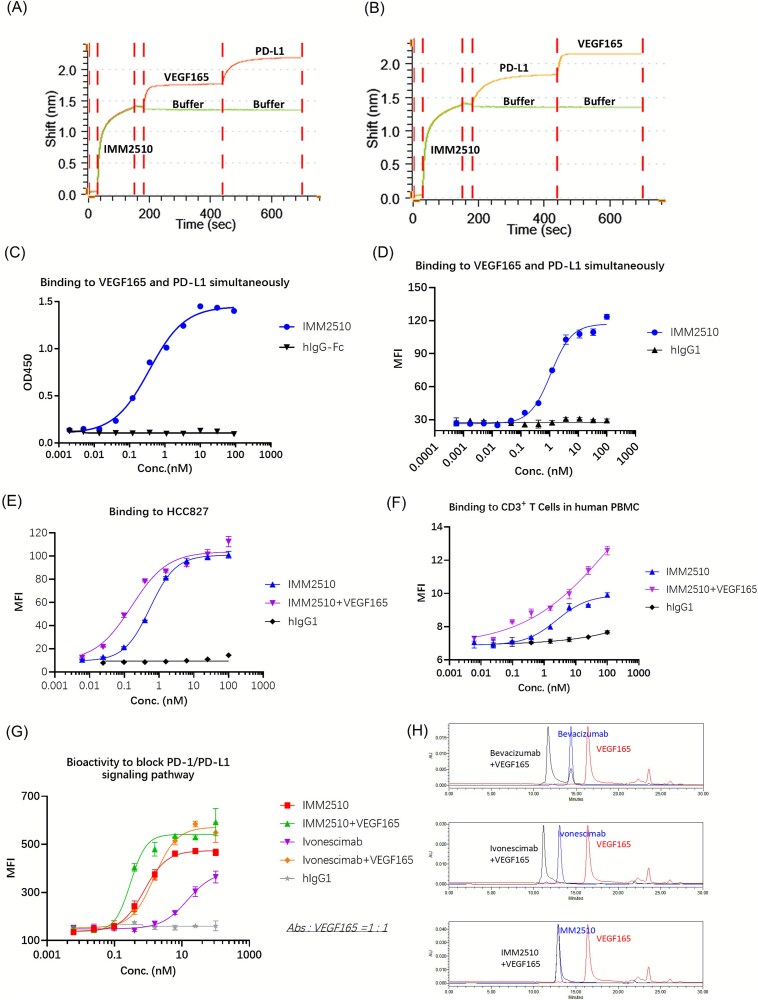
VEGF165 enhances the binding and blocking activity of IMM2510 toward PD-L1. (a, b) simultaneous binding kinetics to VEGF165 and PD-L1, analyzed by gator bio. (c) Simultaneous binding to immobilized PD-L1 and VEGF165, measured by ELISA; (d) simultaneous binding to cell-surface PD-L1 (Raji-PD-L1) and VEGF165, measured by flow cytometry; (e, f) VEGF165 enhances IMM2510 binding to PD-L1 on HCC827 cells and CD3^+^ T cells; (g) VEGF165 enhances IMM2510 blockade of PD-1/PD-L1 signaling in Jurkat-NFAT-eGFP-PD1 reporter assay; (h) SEC-HPLC showing IMM2510 does not form large complexes with dimeric VEGF165;

Figure 4a and b demonstrate that IMM2510 simultaneously engages PD-L1 and VEGF165. Moreover, Independent assays on plates and cells confirmed concurrent dual-target engagement (Fig. 4c and d). The presence of VEGF165 increased the apparent binding of IMM2510 to PD-L1 on HCC827 tumor cells (Fig. 4e) and on CD3^+^ T cells in human PBMCs (Fig. 4f). In the Jurkat-PD-1/Raji-PD-L1 NFAT reporter system, pre-binding IMM2510 with VEGF165 elicited a stronger rescue of the NFAT signal (Fig. 4g and Supplementary Fig. S3a). Similarly, ivonescimab also resulted in a pronounced enhancement, indicating a cooperative effect. Notably, IMM2510 appeared less dependent on the presence of VEGF than ivonescimab. This VEGF-enhanced PD-L1 engagement suggests a cooperative binding mechanism for IMM2510, which may contribute to its antitumor efficacy.

Furthermore, ivonescimab and bevacizumab have been previously reported to form soluble large complexes with dimeric VEGF [20], potentially leading to Fc domain multimerization and platelet activation [26]. We assessed complex formation of IMM2510 by SEC-HPLC. Under identical conditions, IMM2510 formed a single complex with dimeric VEGF165, whereas bevacizumab and ivonescimab formed immune complexes that eluted as larger-MW peaks (Fig. 4h).

### IMM2510 exhibits antitumor efficacy *in vivo*

**Figure 5:**
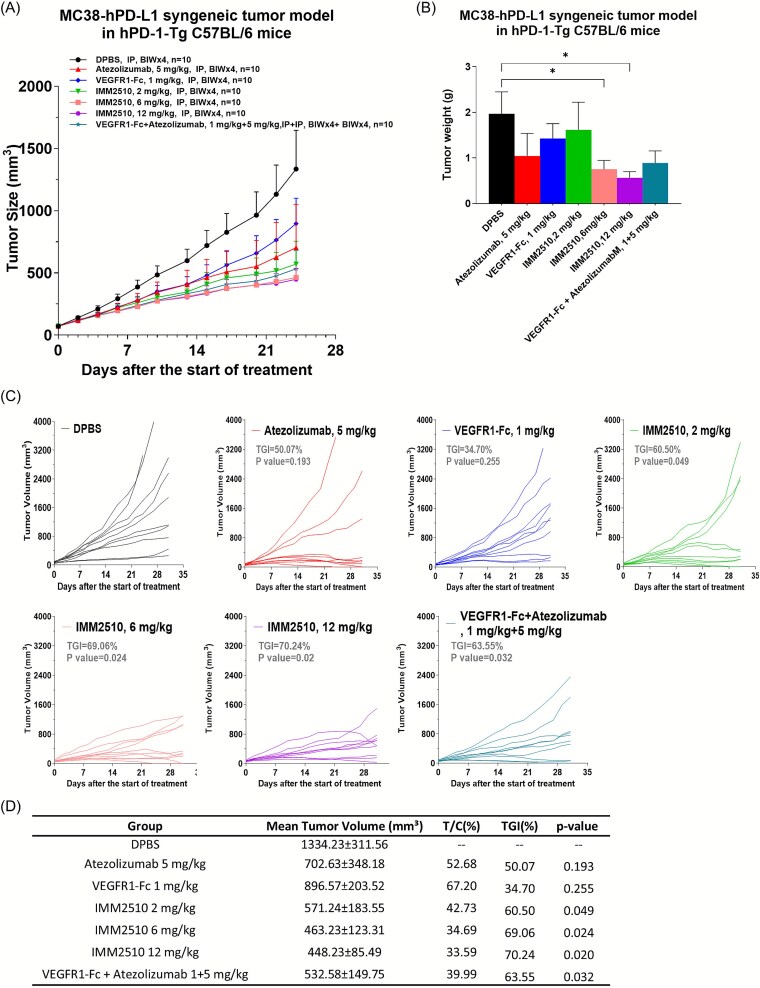
Anti-tumor efficacy of IMM2510 in MC38-hPD-L1 syngeneic tumor model. (a) Tumor growth curves in hPD-1-Tg C57BL/6 mice treated with atezolizumab, VEGFR1-fc, IMM2510 and VEGFR1-fc plus atezolizumab; (b) MC38-hPD-L1 tumor weight at the end point; (c) individual tumor growth curves; (d) mean tumor volume and TGI on day 24. ^*^P < .05, ^**^P < .01 vs. DPBS control.;

The efficacy of IMM2510 was evaluated in hPD-1 transgenic C57BL/6 mice bearing MC38-hPD-L1 syngeneic tumors. Based on our preliminary *in vivo* dose-finding in the Raji-PDL1 xenograft tumor model in CB17-SCID mice, Animals were randomized to receive vehicle, atezolizumab at 5 mg/kg, VEGFR1-Fc at 1 mg/kg, IMM2510 at 2, 6, or 12 mg/kg, or the combination of VEGFR1-Fc with atezolizumab. IMM2510 treatment resulted in dose-dependent TGI, with the 12 mg/kg group achieving the greatest effect (~70.24% TGI). Importantly, high-dose IMM2510 outperformed both monotherapies and showed a trend toward superior efficacy compared to the VEGFR1-Fc plus atezolizumab combination (Fig. 5). These findings highlight the therapeutic advantage of integrating dual-targeting activities within a single molecular, which provides superior activity to either parental agent.

**Figure 6:**
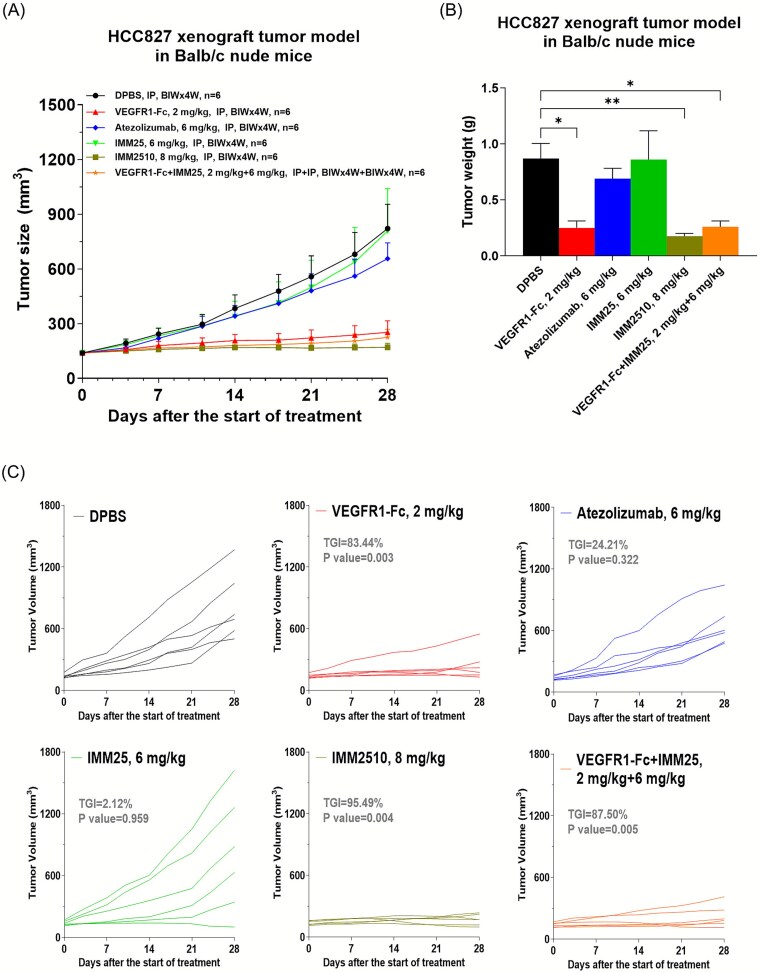
Anti-tumor efficacy of IMM2510 in HCC827 xenograft tumor model. (a) Tumor growth curves in Balb/c nude mice treated with VEGFR1-fc, atezolizumab, IMM25, IMM2510 and VEGFR1-fc plus IMM25; (b) HCC827 tumor weight at the end point; (c) individual tumor growth curves, ^*^P < .05, ^**^P < .01 vs. DPBS control;

To confirm these observations in a human tumor xenograft system, we further tested IMM2510 in human tumor xenograft models. In the PD-L1-expressing HCC827 non-small cell lung cancer model, twice-weekly dosing with IMM2510 led to pronounced tumor regression in a subset of mice and significantly delayed tumor progression across the cohort (Fig. 6a and b). IMM2510 demonstrated superior tumor growth inhibition compared to VEGFR1-Fc combined with IMM25, underscoring the benefit of dual-targeting within a single molecule (Fig. 6c). Consistent with these findings, in the PD-L1-positive MDA-MB-231 triple-negative breast cancer model (Supplementary Fig. S4), IMM2510 again achieved significant tumor growth suppression, outperforming atezolizumab, VEGFR1-Fc, and their combination. Collectively, these results extend the antitumor efficacy of IMM2510 beyond colon and lung cancer models to breast cancer, reinforcing the broad applicability of concurrent PD-L1 blockade and VEGF pathway inhibition.

### Proposed mechanism of action

**Figure 7:**
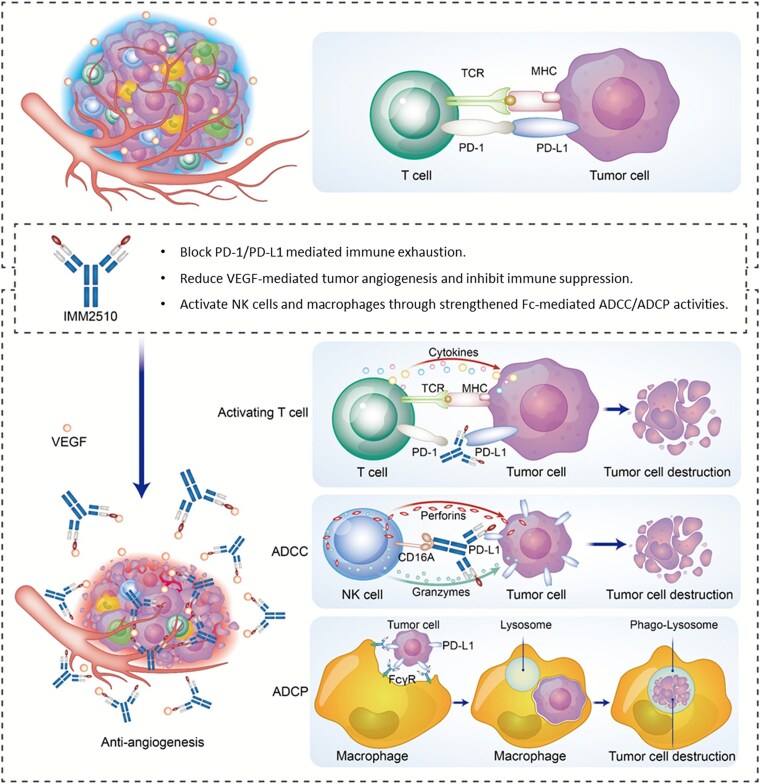
Mechanism of action of IMM2510. IMM2510 is a bispecific antibody targeting both VEGF and PD-L1, constructed by fusing VEGFR1 domain 2 to the N-terminus of each anti-PD-L1 heavy chain. It acts through four key mechanisms: (1) blocking VEGF inhibits angiogenesis and VEGF-mediated immunosuppression in the TME. (2) blocking PD-L1 relieves T-cell inhibition, promoting activation and cytokine release. (3) its engineered fc domain enhances NK cell-mediated ADCC against PD-L1^+^ cells. (4) it promotes macrophage-mediated ADCP.

The collective data support a multifaceted mechanism of action for IMM2510 (Fig. 7). By simultaneously blocking PD-1/PD-L1 and VEGF/VEGFR signaling, IMM2510 relieves immune checkpoint-mediated T-cell suppression while inhibiting tumor angiogenesis and VEGF-driven immunosuppression. The engineered Fc domain confers potent ADCC activity against PD-L1-expressing tumor and stromal cells, directly reducing immunosuppressive populations. Furthermore, the cooperative binding phenomenon, whereby VEGF engagement enhances PD-L1 binding and functional blockade, may provide an additional layer of efficacy unique to this molecular design. Together, these mechanisms contribute to the antitumor activity observed with IMM2510 in preclinical models.

## Discussion

The preclinical data presented here establish IMM2510 as a novel bispecific antibody with the capacity to simultaneously inhibit the PD-1/PD-L1 and VEGF/VEGFR signaling pathways. By integrating immune checkpoint blockade with angiogenesis inhibition, IMM2510 addresses two complementary and clinically validated mechanisms of tumor immune evasion.

Importantly, its Fc region is engineered to enhance ADCC, conferring the ability to selectively deplete PD-L1-expressing tumor and stromal cells—an attribute not shared by currently approved PD-L1 antibodies or other PD-(L)1 × VEGF bispecifics in clinical development. Indeed, avelumab, an approved ADCC-competent anti-PD-L1 antibody, provides a clinical precedent supporting the incorporation of ADCC into anti-PD-L1 modalities [27–29]. The Fc-enhanced ADCC activity differentiates IMM2510 from other molecules in the PD-L1 × VEGF class. Whereas atezolizumab [30] and its bispecific derivatives such as BNT327/PM8002 and HB0025 [31] are Fc-silent, IMM2510 effectively harnesses innate immune effector cells to mediate direct cytotoxicity against PD-L1-expressing tumor. This feature is particularly relevant in tumors characterized by PD-L1 expression within the microenvironment, where direct depletion of suppressive cell populations may complement checkpoint blockade and anti-angiogenesis. Preclinical ADCC assays and the Raji-PDL1 xenograft *in vivo* efficacy model confirmed that IMM2510 induced significantly greater NK cell-mediated killing than IMM25-N297A, an Fc-silent anti-PD-L1 antibody, validating the rationale for this Fc-engineering.

VEGF is a key angiogenic factor and immunomodulator in the TME [32]. Overexpression of VEGF and VEGF/VEGFR signaling promotes pathologic angiogenesis [33]. The combination of PD-1/PD-L1 inhibitors and anti-VEGF drugs has demonstrated improved effectiveness of immunotherapy [34]. The dual-target binding and blockade functions of IMM2510 were confirmed in protein- and cell-based assays, with no impairment of parental binding affinities despite the engineered structure. Functional assays demonstrated robust inhibition of PD-1/PD-L1 signaling and VEGF-driven endothelial proliferation, validating the preservation of both arms of activity. Furthermore, the cooperative binding phenomenon, whereby VEGF165 engagement increased PD-L1 binding and functional blockade, represents a novel mechanistic property that may amplify therapeutic potency in the TME. This cooperative effect could provide IMM2510 with pharmacodynamic advantages over conventional bispecific or combination approaches, warranting further investigation.

IMM2510 engages VEGF165 without forming the higher-order immune complexes characteristic of bevacizumab or Ivonescimab, suggesting a distinct VEGF-binding topology and a lower propensity for Fc multimerization [20, 26]. We speculate this stems from the use of the VEGFR1D2 domain in IMM2510, which differs from bevacizumab. Additionally, VEGF165 binding does not impair ADCC or ADCP activities (Supplementary Fig. S3b and c), and PD-L1 engagement on Raji-PD-L1 cells does not diminish the inhibition of VEGF/VEGFR2 signaling (Supplementary Fig. S3d).

*In vivo* efficacy studies provide compelling evidence of IMM2510’s therapeutic potential. Given that IMM2510 is a dual-targeting PD-L1 × VEGF bispecific, we intentionally selected *in vivo* models that allow assessment of (i) immune checkpoint–dependent activity and (ii) VEGF-driven angiogenesis/TME modulation, recognizing that no single model can fully capture both. Specifically, hPD-1-Tg C57BL/6 mice were employed for the MC38-hPD-L1 syngeneic model, because this setting preserves a fully functional immune system, enabling evaluation of PD-L1 pathway blockade on T-cell activation and immune-mediated antitumor efficacy, as well as potential synergy with VEGF inhibition in an immunocompetent tumor microenvironment. In parallel, Balb/c nude mice were used for the HCC827 and MDA-MB-231 xenograft model to support robust growth of human tumor xenografts without immune rejection, providing a practical platform to interrogate VEGF-mediated angiogenesis and related tumor microenvironment features. hPD-1-Tg C57BL/6 mice support the growth of syngeneic MC38 tumors within a fully functional immune system, enabling the evaluation of immune checkpoint inhibition on T-cell activation and anti-tumor activity. In contrast, the immunocompromised Balb/c nude mice, which lack immune rejection, were utilized to study VEGF-mediated angiogenesis and immunosuppression in the tumor microenvironment.

In the syngeneic MC38-hPD-L1 model, IMM2510 demonstrated dose-dependent tumor growth inhibition, achieving superior efficacy compared with monotherapies and even the combination of VEGFR1-Fc with atezolizumab. Similarly, in the HCC827 NSCLC xenograft model, IMM2510 outperformed the VEGFR1-Fc plus PD-L1 antibody combination, leading to marked tumor regression and delayed progression. These results indicate that a single bispecific molecule may provide functional advantages over the pharmacologic mixture of its parental components, potentially reflecting optimized pharmacokinetics, synchronized target engagement, and cooperative binding effects.

PD-(L)1 × VEGF bispecifics such as ivonescimab and BNT327/PM8002 have already demonstrated promising clinical activity in multiple tumor settings, underscoring the viability of this therapeutic class. However, IMM2510 distinguishes itself as, to our knowledge, the only molecule in this category to combine trapping of several VEGF isoforms (VEGF-A, B and PlGF), PD-L1 checkpoint blockade, and Fc-enhanced effector function (Table 1). First, IMM2510 employs a VEGFR1-D2–based ligand-trap to neutralize VEGF, whereas ivonescimab and BNT327/PM8002 utilize an anti-VEGF arm derived from bevacizumab, representing distinct VEGF-binding modalities and molecular architectures. Second, IMM2510 is a PD-L1–targeting IgG1-based bispecific with an engineered Fc (S298A/E333A/K334A) designed to enhance FcγR engagement, and we demonstrate potent ADCC and ADCP against PD-L1–expressing tumor cells in vitro. In addition, in our comparative assay IMM2510 shows stronger PD-1/PD-L1 signaling inhibitory activity than ivonescimab (Fig. 4g). Mechanistically, PD-L1 binding may also facilitate localization of the antibody to PD-L1–expressing cells within the tumor microenvironment, enabling more effective engagement of Fc-mediated effector functions while simultaneously blocking VEGF signaling. This design offers a differentiated profile that may translate into improved antitumor efficacy, particularly in patients with immunologically “cold” tumors where direct depletion of PD-L1-positive stromal elements could complement T-cell reinvigoration.

**Table 1:** Comparison of IMM2510, Ivonescimab, and PM8002.

	IMM2510	Ivonescimab/AK112	BNT327/PM8002
Target	PD-L1 × VEGF	PD-1 × VEGF	PD-L1 × VEGF
Platform	mAb-Trap	mAb-scFv	mAb-VHH
Molecular weight	~169 kDa	~200 kDa	~175 kDa
Fc mediated effect	ADCC/ADCP	No	No
VEGF blocker	VEGFR1-D2	Bevacizumab	Bevacizumab
VEGF binding target	VEGF-A, VEGF-B, and PlGF	VEGF-A	VEGF-A
PD-1/PD-L1 blocker	PD-L1 mAb	PD1-scFv (14C12)	PD-L1-VHH

Taken together, these findings support IMM2510 as a potential next-generation PD-L1 × VEGF bispecific antibody, offering mechanistic and pharmacologic advantages beyond both conventional ICIs and current bispecific competitors. The cooperative binding phenomenon provides a unique mechanistic rationale for its observed potency, while the ADCC-enhanced Fc design represents a critical differentiating feature with the potential to augment clinical efficacy. These data provide a strong foundation for the ongoing clinical evaluation of IMM2510 in solid tumors, including frontline non-small cell lung cancer.

Building on this preclinical and pharmacodynamic rationale, IMM2510 is advancing through China Phase 1/2 with an RP2D of 20 mg/kg Q2W and an active global program including a U.S. Phase 1 initiation. In China, objective response has been observed with monotherapy across different types of solid tumor in phase 1 study, especially in squamous NSCLC, ORR of 35.3% and DCR of 76.5% reported in WCLC 2025 with well tolerated safety profile [35, 36]. The Phase 2 study of IMM2510 in combination with platinum-based chemotherapy to treat first-line NSCLC patients is ongoing. Forthcoming clinical readouts will be pivotal to confirm translatability across populations and to benchmark differentiation versus standard PD-(L)1^+^ chemotherapy.

## Conclusions

IMM2510 is a differentiated PD-L1 × VEGF bispecific antibody that integrates four complementary mechanisms of action: checkpoint blockade, VEGF ligand trapping, Fc-enhanced ADCC, and cooperative binding. Preclinical studies demonstrated potent and consistent antitumor efficacy across syngeneic and xenograft models, including NSCLC and TNBC, with superior activity compared to parental monotherapies or their combinations. By simultaneously activating immunity, inhibiting angiogenesis, and directly depleting PD-L1-expressing cells, IMM2510 represents a mechanistically robust strategy to overcoming resistance to conventional immunotherapy. These findings support its continued development as a promising next-generation therapeutic with broad potential in solid tumors.

## Supplementary Material

Supplemental_Figure_S1_tbag002

Supplemental_Figure_S2_tbag002

Supplemental_Figure_S3_tbag002

Supplemental_Figure_S4_tbag002

Supplemental_Figure_caption_tbag002

## Data Availability

The original contributions presented in the study are included in the article/Supplementary Material. Further inquiries can be directed to the corresponding author.

## Animal research statement

The animal studies were approved by the Institutional Animal Care and Use Committee of WuXi Biologics Co., Ltd. The study was conducted in accordance with the local legislation and institutional requirements.
