# Supplementary figures and images for: IMM2510, a novel anti-PD-L1/VEGF bispecific antibody for cancer immunotherapy
Source: Antib Ther. 2026 Jan 15;9(1):86–99. doi: 10.1093/abt/tbag002 (PMC12967328; doi:10.1093/abt/tbag002)

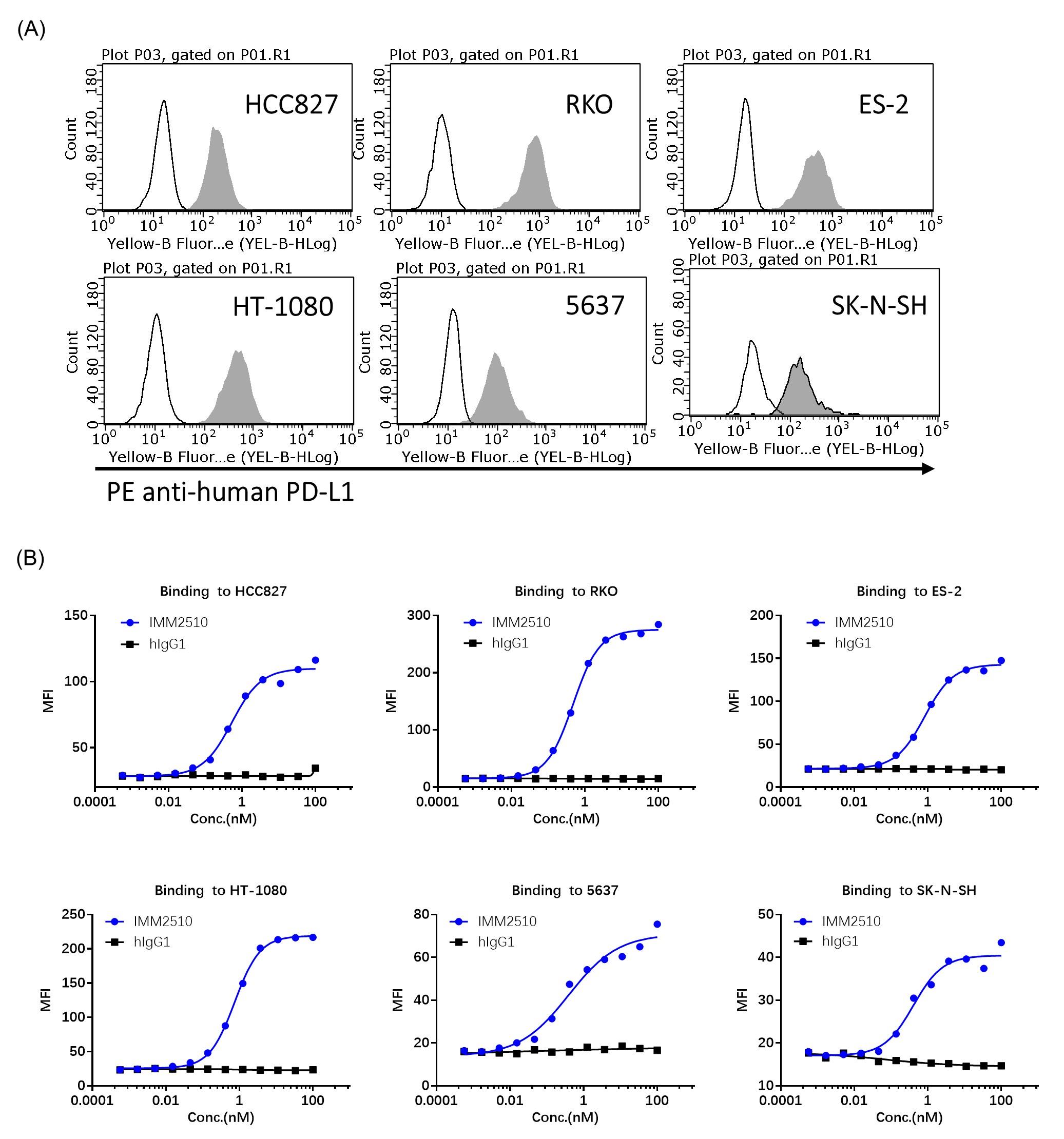

Supplement: Supplemental_Figure_S1_tbag002 [file supplemental_figure_s1_tbag002.jpeg]

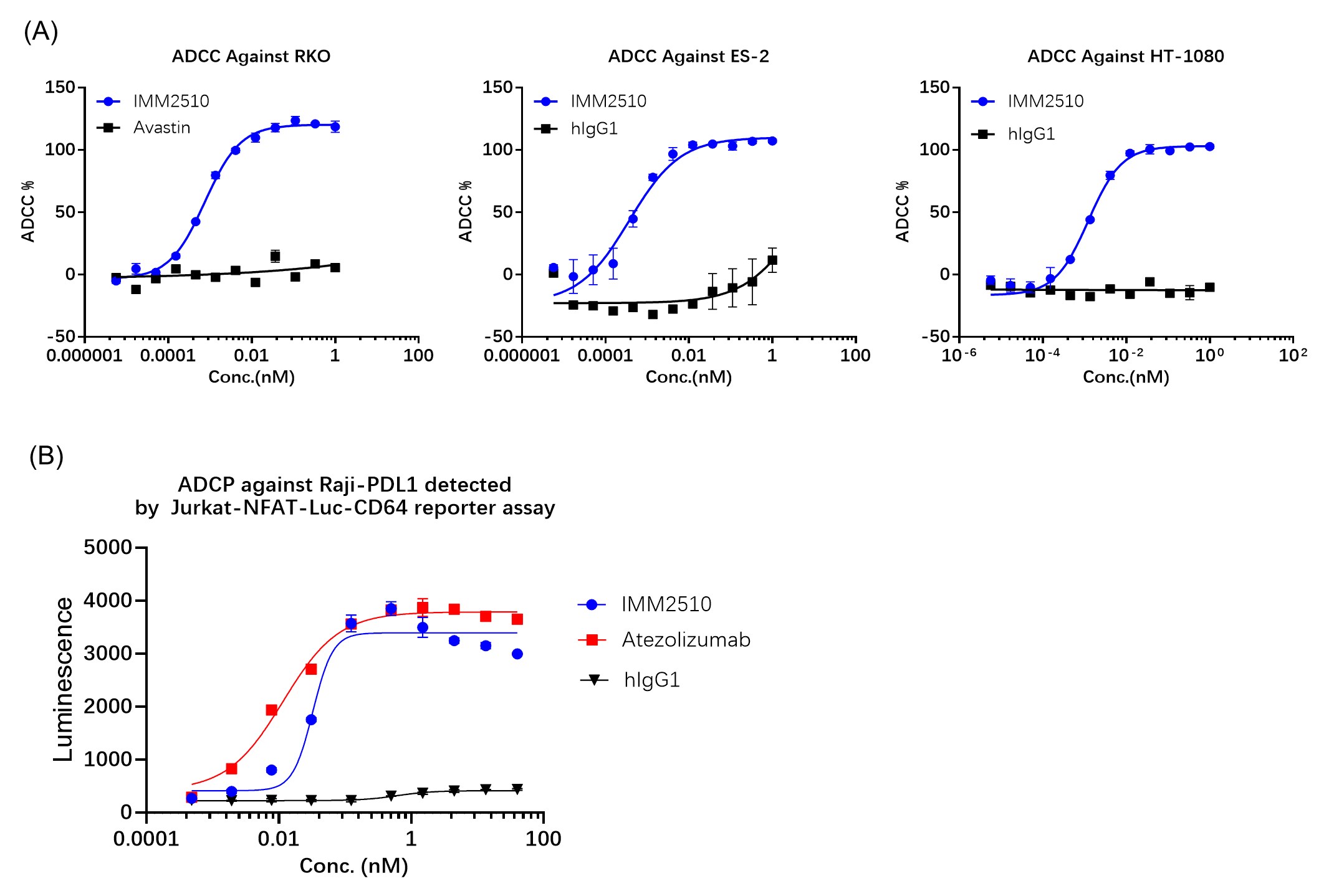

Supplement: Supplemental_Figure_S2_tbag002 [file supplemental_figure_s2_tbag002.jpeg]

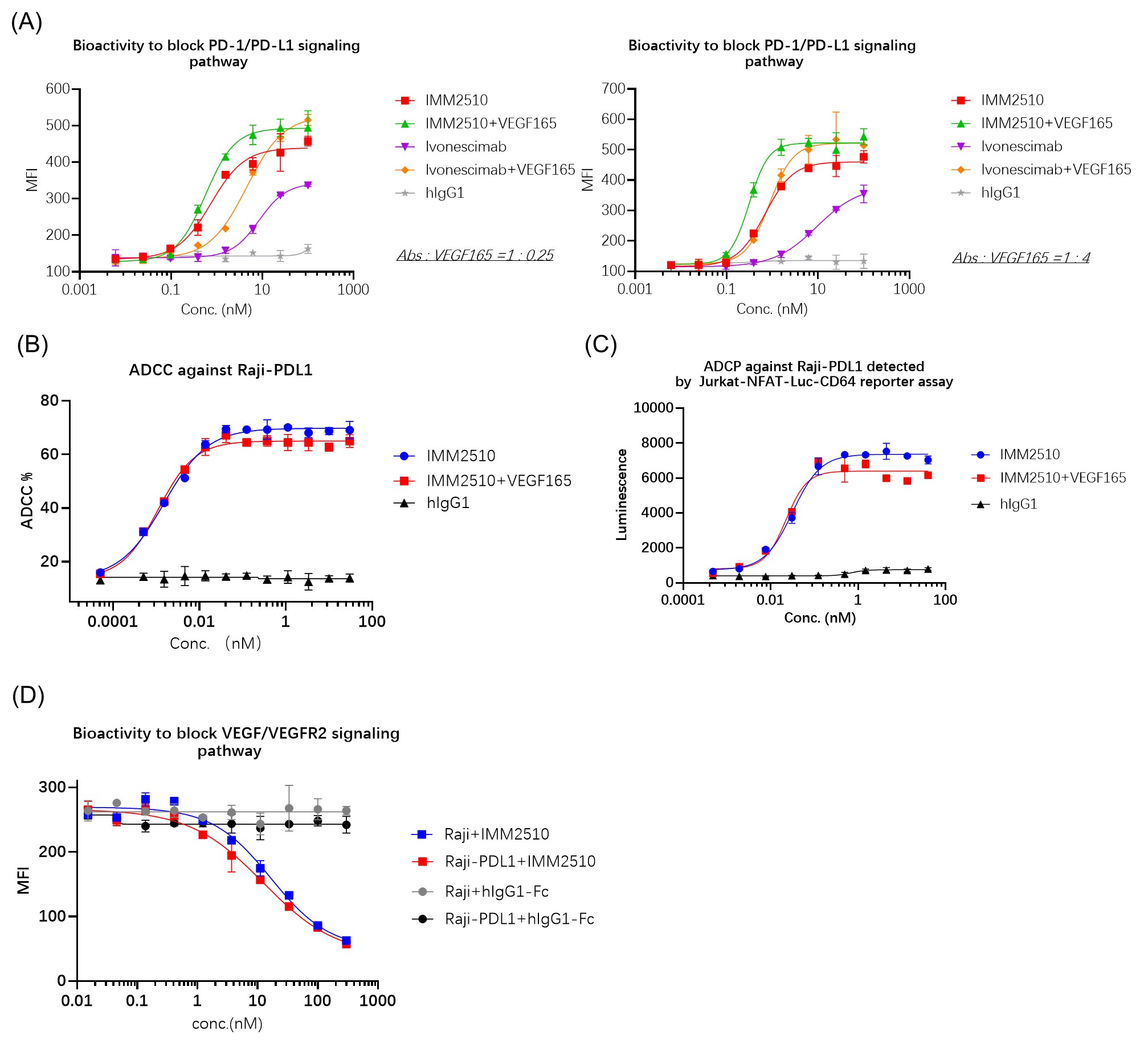

Supplement: Supplemental_Figure_S3_tbag002 [file supplemental_figure_s3_tbag002.jpeg]

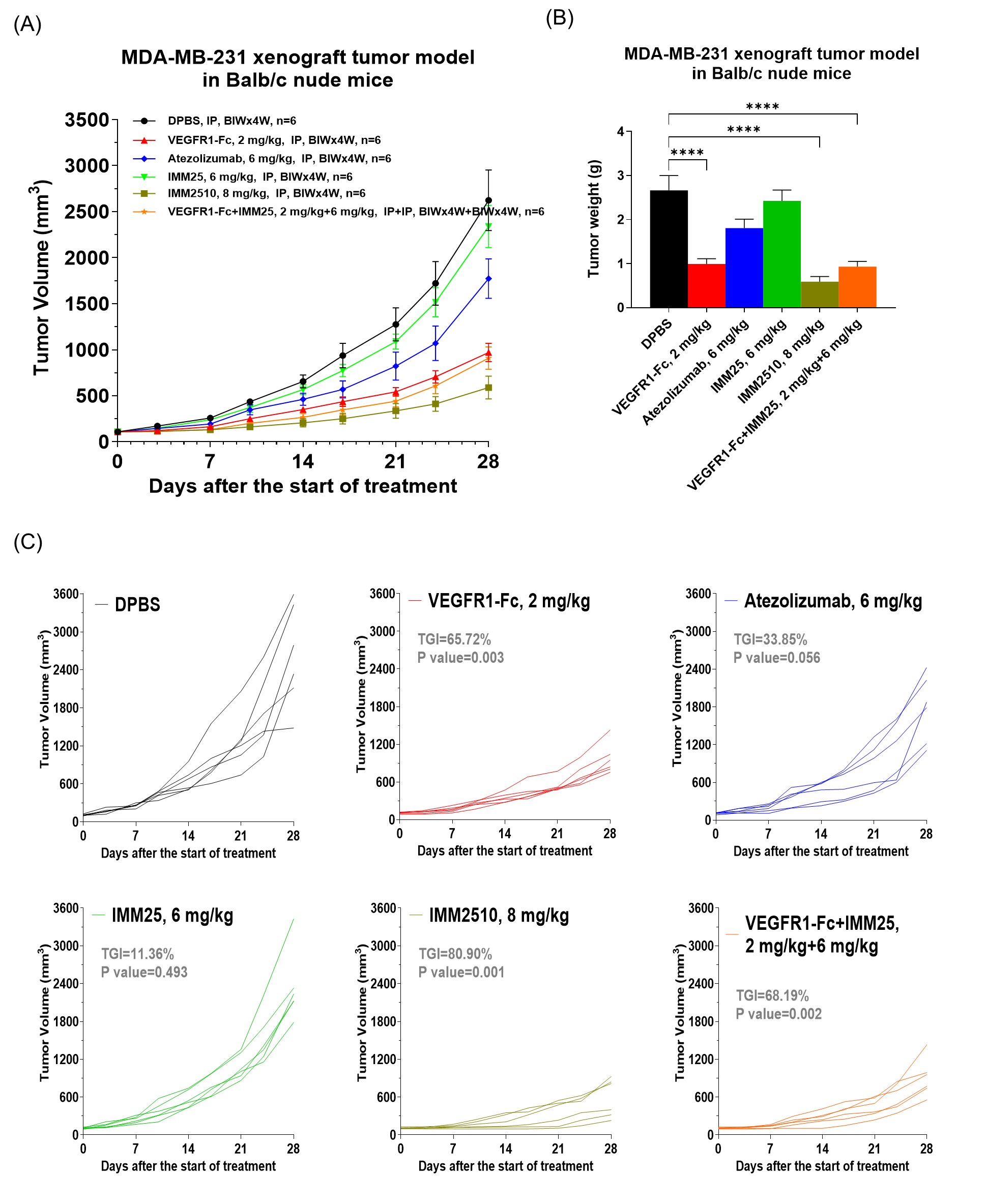

Supplement: Supplemental_Figure_S4_tbag002 [file supplemental_figure_s4_tbag002.jpeg]
