# Supplementary material for: IMM2510, a novel anti-PD-L1/VEGF bispecific antibody for cancer immunotherapy
Source: Antib Ther. 2026 Jan 15;9(1):86–99. doi: 10.1093/abt/tbag002 (PMC12967328; doi:10.1093/abt/tbag002)
Supplement: Supplemental_Figure_caption_tbag002 [file supplemental_figure_caption_tbag002.docx]

## **Supplemental Figure S1.** Binding of IMM2510 to PD-L1^+^ tumor cells; (A) PD-L1 expression levels on various tumor cell lines (HCC827, RKO, ES-2, HT-1080, 5637, SK-N-SH); (B) Binding of IMM2510 to the PD-L1^+^tumor cells, determined by flow cytometry;

## **Supplemental Figure S2**. Fc-mediated ADCC and ADCP activities of IMM2510. (A) ADCC activities against RKO, ES-2 and HT-1080 cells co-cultured with FcγRIIIA (158V)-NK92MI for24 h, measured by Cell Counting Kit-8; (B) ADCP activities measured by reporter gene assay using Jurkat-NFAT-Luc-CD64 reporter cells co-cultured with Raji-PDL1 cells;

## **Supplemental Figure S3**. VEGF165 binding does not impair IMM2510 effector functions or VEGF blockade. (A) VEGF165 enhances IMM2510 blockade of PD-1/PD-L1 signaling in Jurkat-NFAT-eGFP-PD1 reporter assay (IMM2510: VEGF165=1:0.25 and 1:4); (B) VEGF165 binding does not affect the ADCC activities of IMM2510 against Raji-PD-L1 cells, measured by flow cytometer; (C) VEGF165 binding does not affect the ADCP activity of IMM2510, measured by a reporter gene assay; (D) PD-L1 binding does not impair IMM2510 the inhibition of VEGF/VEGFR2 signaling, measured by 293T-NFAT-eGFP-VEGFR2 reporter assay;

## **Supplemental Figure S4.** Anti-tumor efficacy of IMM2510 in MDA-MB-231 xenograft tumor model. (A) Tumor growth curves in Balb/c nude mice treated with VEGFR1-Fc, Atezolizumab, IMM25, IMM2510 and VEGFR1-Fc plus IMM25; (B) MDA-MB-231 tumor weight at the end point; (C) Individual tumor growth curves, * *p* ≤ 0.05; ** *p* ≤ 0.01; *** *p* ≤ 0.001; **** *p* ≤ 0.0001 vs. DPBS control;
